# Semi-parametric risk prediction models for recurrent cardiovascular events in the LIPID study

**DOI:** 10.1186/1471-2288-10-27

**Published:** 2010-04-01

**Authors:** Jisheng Cui, Andrew Forbes, Adrienne Kirby, Ian Marschner, John Simes, David Hunt, Malcolm West, Andrew Tonkin

**Affiliations:** 1 World Health Organization Collaborating Centre for Obesity Prevention, Deakin University, Melbourne, Australia; 2 Department of Epidemiology and Preventive Medicine, Monash University, Melbourne, Australia; 3 NHMRC Clinical Trials Centre, University of Sydney, Sydney, Australia; 4 Department of Statistics, Macquarie University, Sydney, Australia; 5 Department of Medicine, University of Queensland, Brisbane, Australia

## Abstract

**Background:**

Traditional methods for analyzing clinical and epidemiological cohort study data have been focused on the first occurrence of a health outcome. However, in many situations, recurrent event data are frequently observed. It is inefficient to use methods for the analysis of first events to analyse recurrent event data.

**Methods:**

We applied several semi-parametric proportional hazards models to analyze the risk of recurrent myocardial infarction (MI) events based on data from a very large randomized placebo-controlled trial of cholesterol-lowering drug. The backward selection procedure was used to select the significant risk factors in a model. The best fitting model was selected using the log-likelihood ratio test, Akaike Information and Bayesian Information Criteria.

**Results:**

A total of 8557 persons were included in the LIPID study. Risk factors such as age, smoking status, total cholesterol and high density lipoprotein cholesterol levels, qualifying event for the acute coronary syndrome, revascularization, history of stroke or diabetes, angina grade and treatment with pravastatin were significant for development of both first and subsequent MI events. No significant difference was found for the effects of these risk factors between the first and subsequent MI events. The significant risk factors selected in this study were the same as those selected by the parametric conditional frailty model. Estimates of the relative risks and 95% confidence intervals were also similar between these two methods.

**Conclusions:**

Our study shows the usefulness and convenience of the semi-parametric proportional hazards models for the analysis of recurrent event data, especially in estimation of regression coefficients and cumulative risks.

## Background

Many clinical and epidemiological cohort studies involve health outcomes that a participant of the study can experience several times during the follow-up period. Such outcomes are often termed recurrent or repeated events [1,2]. For example, transient ischemic attacks can occur repeatedly among patients with cerebrovascular disease [3]. A person who is infected with HIV may experience several opportunistic infections in HIV/AIDS clinical trials [4]. However, traditional statistical methods for the analysis of cohort study data have been focused on the first occurrence of an outcome [5]. It is reasonable to assume that the first occurrences of an outcome among different individuals are independent, which is an important assumption in modelling these events.

However, the methods for the analysis of first events are inefficient in the analysis of recurrent event data because of the possible correlation among recurrent outcomes in an individual. An individual may be prone to develop more recurrent events or the time interval between these events may be shorter than others in the study depending on the characteristics of the individual. Therefore, the assumption of independence of the event times in the first event analysis method is often violated for recurrent event data. Updated analytical methods are needed to account for the dependence of the repeated measurements in a person during follow-up study.

Various statistical methods have been proposed for the analysis of recurrent event data [6-9]. The conditional models usually define different risk sets for different recurrent events using stratification techniques [8,10]. The gap time between two consecutive recurrent events is usually used in a conditional model. The conditional model can be used to estimate the rate of a subsequent event given an individual has already had such an event during follow-up. On the other hand, Cox-type regression models have been considered by Prentice et al [11] and Andersen and Gill [12] in their seminal studies in this area. Wei et al [13] proposed a novel semi-parametric proportional hazards model and Lin [14] presented general theory for this method. Semi-parametric models have further been updated and applied by Pepe and Cai [15] for time-dependent covariates, by Lawless and Nadeau [16] for a robust method, and by Glidden and Vittinghoff [17] for analyzing multicenter clinical trial data. Wang and Chang [18] clarified two sampling designs in a longitudinal study and devoted their method to the design where the initial occurrence of an event is used as the enrolment criterion and repeated occurrences of the same event are observed during follow-up. Pena et al [19] and Pena et al [20] also discussed the general class of semi-parametric models for recurrent events. Metcalfe and Thompson [21] reviewed and evaluated the semi-parametric model method in the analysis of recurrent event data and concluded that the application of this method to recurrent event data is justified.

The aim of this study was to apply several semi-parametric models to the analysis of recurrent myocardial infarction (MI) events in a large randomized placebo-controlled clinical trial in Australia and New Zealand [22,23]. We focused on investigating the significant risk factors for both the first and recurrent MI events during follow-up and examining whether there was significant difference in their effects for the first and recurrent MI events.

## Methods

### The LIPID study

The Long-term Intervention with Pravastatin in Ischaemic Disease (LIPID) study [22-24] was a double-blinded randomized placebo-controlled trial of a cholesterol-lowering drug, pravastatin (40 mg daily). The study was undertaken in 9014 patients who were 31 to 75 years of age at the time of randomization. They had a history of myocardial infarction or hospitalization for unstable angina 3-36 months previously and initial plasma total cholesterol levels of 155 to 271 mg per deciliter (4.0 - 7.0 mmol/liter) and triglyceride levels < 445 mg/dl (5 mmol/L). Patients were followed up for a median of 6.0 years between 1990 and 1997. In the present analysis, data from the 8557 patients (4286 randomized to receive pravastatin and 4271 to receive placebo) who did not have any missing baseline information were used, which is consistent with a previous study [24]. The primary pre-specified endpoint in the LIPID study was coronary heart disease (CHD)-related death and for secondary analyses, a composite of CHD death or non-fatal myocardial infarction. Details of the study design have been published previously [22,23].

The outcome variables in this present analysis were the recurrence of an MI event during follow-up and the time from randomization to each MI event. Explanatory variables included gender (1 for males and 0 for females), age at randomization (centered at 60 years for ease of computing the baseline hazard rate), smoking status (1 for smokers and 0 for non-smokers), total serum cholesterol (centered at 5.0 mmol/l), high density lipoprotein (HDL)-cholesterol (centered at 1.0 mmol/l), treatment group (1 for pravastatin assignment and 0 for placebo), qualifying acute coronary syndrome (ACS) at baseline (unstable angina, single MI, or multiple MI before randomization), coronary revascularization (never, before the qualifying event, or after the qualifying event but before randomization), dyspnea grade (0 for NYHA < III and 1 for NYHA ≥ III) based on New York Heart Association definition [25], angina grade (0 for CCVS < III and 1 for CCVS ≥ III) based on the Canadian Cardiovascular Society definition [26], duration of angina (0 for ≤5 years and 1 for >5 years), use of aspirin (0 for no and 1 for yes), country of residence (Australia or New Zealand), and history of stroke, diabetes mellitus or hypertension. Interactions between these risk factors were also examined in the analysis.

The following multivariate regression models were fitted to the data to incorporate all the above mentioned explanatory variables. The backward selection method was used to select the final model which includes risk factors which were statistically significant at the 0.05 level.

### Semi-parametric model

The semi-parametric model considered in this article is an extension of the Cox proportional hazards model [5]. Suppose there are *n *participants in the study. For the *i*th person (*i *= 1, 2, ..., *n*), the model is of the following form(1)

where  is the general hazard function and  is the baseline hazard function at time *t *for the *k*^th ^recurrent event *(k = 1, ..., K)*. The analysis time *t *is calculated from the date of randomization to the date of each recurrent MI event for each person in the study. Both the risk factor *x *and the associated regression coefficient *β*^(*k*) ^are (*p*+1)-dimensional vectors, where *p *is the number of covariates in the model. Parameter *β*^(*k*) ^is estimated using the partial likelihood method [27]. The advantage of using the semi-parametric model is that it does not require explicit specification of the dependence structure between the times of recurrent events of each person; instead it uses the robust Huber/White sandwich estimator to obtain the variance of the estimated regression coefficients [28-30]. The robust variance estimator can be easily calculated using most of the standard statistical software packages. The cumulative risk of a specific MI event after randomization can be calculated by

where  is the cumulative hazard by time *t *[31].

The censoring mechanism was assumed to be independent of the recurrent event process, as it is in most standard time-to-event analyses. A person was censored if he/she died of non-cardiovascular causes, or was lost to follow-up or was alive at the end of the study. Therefore, a person might have several recurrent MI events, or might not experience any MI event before being censored. Multiple data records might be observed for each person for possible occurrence of an MI event or occurrence of censoring. However, the occurrence of an MI was assumed not to influence the occurrence of censoring, and vice versa, in the present analysis.

### Model comparison

Several semi-parametric models were fitted to the LIPID study data. Model 1 assumes that the covariate effect *β*^(*k*) ^and the baseline hazard rate  were different for the first and subsequent MI events. Model 2 assumes that the covariate effects *β*^(*k*) ^were the same for the first and subsequent MI events but the baseline hazard rates were different for these recurrent events. Model 3 assumes that the covariate effects *β*^(*k*) ^were different for the recurrent events but the baseline hazard rates  were the same. Model 4 assumes that both the covariate effects *β*^(*k*) ^and the baseline hazard rates  were the same for the recurrent events.

### Model fitting

Appropriate data formats were prepared for fitting a semi-parametric model. No matter how many observed data records that a person can have, a total of *K *records were created for each person in the study, where *K *was the maximum number of recurrent events that were experienced by all participants in the study. For a person who had an MI event at time *t*, the value of the corresponding outcome was 1 for this time point. After the time of the final MI event, the value of the outcome was assumed to be 0 in order to make a total of K data records. In addition, an indicator variable was created for each data record of a person in the study. Details concerning preparation of appropriate data formats can be found in Therneau and Grambsch [8]. The statistical analyses in this article were conducted using Stata software version 10 [32], which allows semi-parametric model analysis to be conducted easily with no specialized programming required. These analyses were based on the intention-to-treat principle where an individual was assumed to take the treatment that was assigned to him or her at the time of randomization [33].

## Results

### Follow-up in the LIPID Study

Table 1 shows the number of MI events and the outcome status of participants at the end of the study. A total of 870 MI events occurred in 745 patients (an average of 1.2 events per person) during follow-up, of whom 313 had been randomized to receive pravastatin and 432 to receive placebo. In all, 7.3% (313/4286) of the persons who were assigned to receive pravastatin and 10.1% (432/4271) assigned to placebo had at least one MI event during follow-up. The former proportion was significantly lower than the latter (*z *= 4.6, *p *< 0.001). The number of persons who had more than one MI event during follow-up was generally low. About 0.9% (37/4286) of the persons in the pravastatin group and 1.3% (56/4271) in the placebo group had more than one MI event during follow-up. Because of the small numbers of participants who had more than two MI events in this study, the following analyses were focused on the first two MI events, i.e. we assumed *K = 2 *in model (1).

**Table 1:** Number of myocardial infarctions and outcome status for the participants in the LIPID study

Variable	Treatment	
	
	Pravastatin	Placebo
Number of MI events		
0	3973	3839
1	276	376
2	31	39
3	3	13
4	2	3
5	1	1
Outcome status		
Died	470	592
Alive and had an MI event	236	305
Alive but no MI event	3580	3374

MI = myocardial infarction

Table 1 also shows that a total of 1062 patients (470 assigned pravastatin and 592 placebo) died during follow-up. Another 541 patients (236 assigned pravastatin, 305 placebo) experienced at least one MI event during follow-up and were still alive at the end of the study. The other 6954 patients (3580 assigned pravastatin, 3374 placebo) did not experience any MI event during follow-up and were still alive at the end of the study.

### Model comparison

Different semi-parametric models are compared in Table 2. The same covariates were selected by all 4 models, which include age, smoking status, total cholesterol and HDL cholesterol levels, qualifying ACS event at baseline, coronary revascularization, history of stroke or diabetes, angina grade and treatment with pravastatin. Model 2 that has the same effect for all recurrent events but with different baseline hazard functions had the smallest AIC and BIC values, suggesting that this model fitted the data better than other three models. Model 1 that has different effects for the recurrent events and different baseline hazard functions had the second smallest AIC and BIC values. The likelihood ratio test showed that there was no significant difference between model 1 and model 2 (χ^2 ^= 6.51, d.f. = 12, p = 0.89). On the other hand, model 4 that has the same effect for the recurrent events and the same baseline function had the largest AIC and BIC values, suggesting that this model had the poorest fit to the data. Therefore, model 2 was considered to be the best model.

**Table 2:** Comparison of different semi-parametric models in the LIPID study

Model	Log-likelihood	**d.f**.	AIC	BIC
Different baseline hazard				
Model 1: Different effect	-7317.331	24	14682.66	14868.61
Model 2: Same effect	-7320.586	12	14665.47	14758.14
Same baseline hazard				
Model 3: Different effect	-7646.675	24	15341.35	15527.29
Model 4: Same effect	-7915.245	12	15854.49	15947.46

AIC = Akaike Information Criterion; BIC = Bayesian Information Criterion; d.f. = degree of freedom

### Same effect model

Table 3 shows the significant risk factors and the estimated hazard ratios and 95% confidence intervals as specified in model 2. Specifically, compared with patients in the placebo group, patients in the pravastatin group had a 29% (95% CI 17 - 39%) reduction in the risk for the both MI events. For one unit (mmol/l) increase in total cholesterol level, the risk of an MI event (both the first and second event) increased by 19% (95% CI 9 - 30%); while for one unit (mmol/l) increase in HDL-cholesterol, the risk of an MI event decreased by 62% (95% CI 46 - 74%). Having a history of stroke increased the risk of an MI event by 47% (95% CI 9 - 99%) and a history of diabetes increased the risk of an MI event by 37% (95% CI 8 - 72%).

**Table 3:** Same effect model of the recurrent MI events in the LIPID study

Risk factor	Hazard ratio	95% CI	P-value
Age (year)	1.02	1.01 - 1.03	<0.001
Smoking status			
Non-smoker	1.0		
Current smoker	1.51	1.20 - 1.90	<0.001
Total cholesterol	1.19	1.09 - 1.30	<0.001
HDL cholesterol	0.38	0.26 - 0.54	<0.001
Qualifying event for ACS			
Unstable angina	1.0		
Single MI	1.29	1.06 - 1.57	0.01
Multiple MI	1.98	1.57 - 2.50	<0.001
Revascularization			
Never	1.0		
Before ACS	1.59	1.26 - 2.01	<0.001
Since ACS	0.70	0.58 - 0.85	<0.001
History of stroke			
No	1.0		
Yes	1.47	1.09 - 1.99	0.01
Diabetes mellitus			
No	1.0		
Yes	1.37	1.08 - 1.72	0.009
Angina grade			
CCVS < III	1.0		
CCVS ≥ III	1.45	1.15 - 1.82	0.001
Treatment			
Placebo	1.0		
Pravastatin	0.71	0.61 - 0.83	<0.001

ACS = Acute Coronary Syndrome; CCVS = Canadian Cardiovascular Society Class of angina; HDL = High Density Lipoprotein; MI = Myocardial Infarction.

Similarly, compared with patients with unstable angina as their qualifying event, the risk for an MI event during follow-up was increased by 29% (95% CI 6 - 57%) in those with a single MI event prior to randomization, while patients with multiple MI events prior to randomization had a 98% (95% CI 57 - 150%) increased risk. Compared with patients without a history of coronary revascularization, those who had revascularization before randomization had a 59% (95% CI 26 - 101%) increased risk for an MI event during follow-up; while patients who had revascularization after the qualifying event and before randomization had a 30% (15 - 42%) decreased risk. Having an angina grade III also increased the risk of an MI event during follow-up by 45% (95% CI 15 - 82%). Smoking also increased the risk of an MI event by 51% (95% CI 20 - 90%).

### Different effect model

Table 4 shows the significant risk factors and the estimates of hazard ratios and 95% confidence intervals as specified in model 1. This model has different covariate effects for the first and second recurrent MI events. Based on the Wald test [34], there was no significant difference in the effects of these risk factors between the first and second MI events (*p-*values were between 0.076 and 0.91 in all cases). For example, compared with patients in the placebo group, patients assigned pravastatin had a 28% (95% CI 17 - 38%) reduction in the risk for the first MI and 34% (95% CI 1 - 57%) reduction in the risk of a second MI. Compared with patients with unstable angina as their qualifying event, patients with multiple MIs prior to randomization had a 92% (95% CI 54 - 140%) increased risk for a first MI event and 146% (95% CI 34 - 354%) increased risk for a second MI during follow-up.

**Table 4:** Different effect model of the recurrent MI events in the LIPID study

Risk factor	First MI event			Second MI event			Wald test^†^
	
	HR	95% CI	P-value	HR	95% CI	P-value	
Age (year)	1.02	1.01 - 1.03	<0.001	1.04	1.01 - 1.06	0.007	0.14
Smoking status							
Non-smoker	1.0			1.0			
Current smoker	1.49	1.20 - 1.85	<0.001	1.71	0.94 - 3.12	0.079	0.63
Total cholesterol	1.18	1.08 - 1.28	<0.001	1.34	1.08 - 1.66	0.007	0.21
HDL cholesterol	0.41	0.29 - 0.59	<0.001	0.17	0.06 - 0.47	0.001	0.076
Qualifying event for ACS							
Unstable angina	1.0			1.0			
Single MI	1.27	1.06 - 1.53	0.011	1.44	0.81 - 2.54	0.21	0.66
Multiple MI	1.92	1.54 - 2.40	<0.001	2.46	1.34 - 4.54	0.004	0.39
Revascularization							
Never	1.0			1.0			
Before ACS	1.54	1.23 - 1.92	<0.001	2.02	1.10 - 3.74	0.024	0.34
Since ACS	0.70	0.58 - 0.85	<0.001	0.65	0.36 - 1.16	0.14	0.76
History of stroke							
No	1.0			1.0			
Yes	1.50	1.12 - 2.00	0.006	1.31	0.57 - 3.04	0.53	0.75
Diabetes mellitus							
No	1.0			1.0			
Yes	1.40	1.12 - 1.75	0.003	1.14	0.58 - 2.23	0.70	0.53
Angina grade							
CCVS < III	1.0			1.0			
CCVS ≥ III	1.44	1.16 - 1.80	0.001	1.48	0.83 - 2.65	0.18	0.91
Treatment							
Placebo	1.0			1.0			
Pravastatin	0.72	0.62 - 0.83	<0.001	0.66	0.43 - 0.99	0.047	0.67

† P-value for Wald test. ACS = Acute Coronary Syndrome; CCVS = Canadian Cardiovascular Society Class of angina; HDL = High Density Lipoprotein; MI = Myocardial Infarction.

Similarly, compared with patients who never had revascularization, patients who had coronary revascularization before their qualifying acute coronary syndrome had 54% (95% CI 23 - 92%) increased risk for a first MI and 102% (95% CI 10 - 274%) increased risk for a second MI during follow-up. Other risk factors (smoking status, history of stroke or diabetes mellitus and angina grade) significantly increased the risk for the first but not the second MI event, probably due to the small number of observed second MI events, but in no case was there evidence of a different effect on first MI compared to a second MI (Table 4).

**Figure 1:**
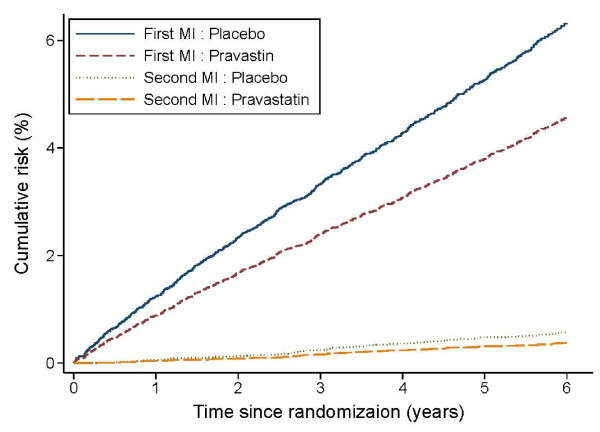
**Estimated baseline cumulative risk of myocardial infarction (MI) during follow-up for a non-smoking patient aged 60 years with total cholesterol level 5.0 mmol/l, HDL cholesterol 1.0 mmol/l, angina grade less than III, with unstable angina as the qualifying acute coronary syndrome and no history of revascularization, stroke or diabetes mellitus before randomization**.

Figure 1 shows the cumulative risk of a specific MI event after randomization under model 2. For ease of drawing this figure, baseline values of all significant categorical variables and centralized values of all significant continuous variables were used in model 2. These values included non-smoking status, 60 years of age, total cholesterol level of 193 mg/dl (5.0 mmol/l), HDL cholesterol level of 39 mg/dl (1.0 mmol/l), angina grade III or less, previous unstable angina as the qualifying acute coronary syndrome at baseline and no history of revascularization, stroke or diabetes mellitus prior to randomization. The cumulative risk of the first MI event within 5 years of randomization for such a person with these baseline values was 3.6% if the person was assigned to pravastatin and 5.1% if assigned to placebo. The cumulative risk for a second MI event within 5 years of randomization was lower but there was a similar relative treatment effect, being 0.5% for pravastatin and 0.7% for placebo.

### Same baseline hazard model

We also fitted a semi-parametric model with the same baseline hazard function for the first and second MI events to the data. Compared with the estimated effects in Tables 3 and 4, the estimated effects under this new model increased slightly for the first MI event; while the estimated effects for the second MI event decreased slightly. The baseline cumulative risk in 5 years after randomization was about 2.8% for patients assigned to receive pravastatin and 3.6% for patients to receive placebo.

### First MI event model

Finally we fitted the traditional proportional hazards model by considering only the first MI event in the analysis, as shown in Table 5. Because the number of observations in this study was only half of those in Table 3 and 4, this model was not comparable directly with those in Tables 3 and 4 using the AIC and BIC methods. However, the same significant explanatory variables in Tables 3 and 4 were found to be statistically significant for this model. The estimated effects of these risk factors were the same as those given in Table 4 for the first MI event.

**Table 5:** First event model of the recurrent MI events in the LIPID study

Risk factor	Hazard ratio	95% CI	P-value
Age (year)	1.02	1.01 - 1.03	<0.001
Smoking status			
Non-smoker	1.0		
Current smoker	1.49	1.20 - 1.86	<0.001
Total cholesterol	1.18	1.08 - 1.29	<0.001
HDL cholesterol	0.41	0.29 - 0.58	<0.001
Qualifying event for ACS			
Unstable angina	1.0		
Single MI	1.27	1.06 - 1.53	0.008
Multiple MI	1.92	1.55 - 2.39	<0.001
Revascularization			
Never	1.0		
Before ACS	1.54	1.24 - 1.90	<0.001
Since ACS	0.70	0.58 - 0.85	<0.001
History of stroke			
No	1.0		
Yes	1.50	1.12 - 2.00	0.007
Diabetes mellitus			
No	1.0		
Yes	1.40	1.12 - 1.74	0.003
Angina grade			
CCVS < III	1.0		
CCVS ≥ III	1.44	1.16 - 1.80	0.001
Treatment			
Placebo	1.0		
Pravastatin	0.72	0.62 - 0.83	<0.001

ACS = Acute Coronary Syndrome; CCVS = Canadian Cardiovascular Society Class of angina; HDL = High Density Lipoprotein; MI = Myocardial Infarction.

### Model Comparison

Compared with our previous analysis results under the parametric frailty model [10], we found that the same explanatory variables were selected into the final model in this study by the same selection procedure. The estimates of the regression coefficients and 95% confidence intervals were also similar. For example, the estimate of the smoking effect was 1.49 (95% CI 1.17 - 1.89) under the parametric frailty model (Table 3 in [10]) compared with 1.51 (95% CI 1.20 - 1.90) under the semi-parametric model (Table 3 in this article). Similarly, the estimate of the treatment effect of pravastatin was 0.71 (95% CI 0.60 - 0.83) under the parametric frailty model compared with 0.71 (95% CI 0.61 - 0.83) under the semi-parametric model.

However, interpretation of the risk of a recurrent event is different between the parametric conditional model and the semi-parametric proportional hazards model. The time period used to calculate the cumulative risk under the parametric conditional model was the time interval between two consecutive recurrent events; while it was from randomization to each recurrent event under the semi-parametric proportional hazards model. For example, under the parametric conditional model (Figure 1 in [10]), the cumulative risk of a second MI event within 5 years was estimated to be 10.3% and 7.6% for persons assigned to placebo and pravastatin, respectively. However, under the semi-parametric proportional hazards model, the corresponding risk was estimated to be 0.7% and 0.5%, respectively. It is noteworthy that the cumulative risk for the first MI event was similar under these two models because the common time origin was used in both methods (5.6% and 4.1% under the parametric conditional model compared with 5.1% and 3.6% under the semi-parametric proportional hazards model).

## Discussion

In this article, we applied several semi-parametric models [i.e., Wei, Lin and Weissfeld (WLW) models] to the analysis of the risk of recurrent myocardial infarctions in the LIPID study. We found that risk factors such as age, smoking status, total cholesterol and HDL cholesterol levels, the nature of the qualifying acute coronary syndrome, coronary revascularization, history of stroke or diabetes, angina grade and treatment with pravastatin were significant in the development of an MI event during follow-up. No significant difference was found in the effects of these risk factors between the first and second MI events. This is consistent with ongoing benefits of pravastatin for patients after they have a first MI while on active treatment. This analysis result is also consistent with a previous study [24], which analyzed data from the first MI and fatal coronary heart disease-related death only.

A major concern about the WLW model is that each individual is considered to be at risk of all recurrent events from the start of the observation period, while the conditional model [11] assumes that an individual is at risk of the k^th ^event only if the person experienced the (k-1)^th ^event. However, the WLW method has been applied to recurrent event data because the nature of the relationship between recurrent events needs not to be known at the commencement of a study [6,14]. Furthermore, treatment effects are estimated based on the comparison of treatment and placebo groups. The WLW method is based on groups comparable at the time of randomization [35,36].

It has been suggested that a fuller picture of the treatment effect can be obtained from the application of both the marginal and conditional models [14,37]. We have published analysis results based on the conditional model [10]. One aim of this article is to compare the results of the marginal model with those from the conditional model.

Comparison of our previous findings with the current analysis shows that the semi-parametric models selected the same explanatory variables compared with the parametric conditional frailty models. The estimates of the regression coefficients and 95% confidence intervals were also similar in the parametric and semi-parametric models. However, interpretation of the risk of a recurrent event is different between the parametric conditional model and the semi-parametric proportional hazards model. Under the parametric conditional model, the risk of a recurrent event is conditional on a person having had an MI event. The time period for calculating this risk is based on the gap between two consecutive recurrent events. This study shows that the chance of having two MI events within 5 years was low among all participants in the LIPID study; while the previous analysis suggested that the chance of having another MI event among a subset of participants who have already had an MI event was moderate.

Both semi-parametric proportional hazards model and the parametric conditional model are useful tools to further explore the biological process of a medical condition, such as different clinical manifestations of coronary atherosclerosis, our most common major health problem. They allow examination of the sequential occurrence of related events over time. The magnitude and direction of the impact of potential risk factors on these multiple events can also be examined using this approach. Additionally, the relative importance of the predictive values of a risk factor can be compared for different events. It is noteworthy that age, total cholesterol and HDL cholesterol were significant predictors of recurrent as well as the first myocardial infarction during follow-up.

The WLW method has been criticized because of its failure to accommodate the ordered nature of the recurrent events, that is, an individual can be at risk for the k^th ^event before the person experienced the (k-1)^th ^event [38]. Essentially the WLW method assumes that the treatment effect on event k will be "carried over" to subsequent events, which may possibly cause biased estimates of the treatment effects for the later events [39]. According to the definition of the risk set of the WLW method, the occurrence of the first event also influences the time spent at risk for subsequent events though there is no direct treatment effect on the later events.

Another common criticism of the WLW method is that there is no simple joint distribution of all event times to satisfy the proportional hazards assumption [38]. Finally, the WLW method has been criticised for its failure to model the within-subject association of the recurrent events directly, which could possibly result in inefficient estimation of the treatment effect. However, this issue is balanced by the easy implementation of the robust variance estimates for any possible model mis-specification.

Pravastatin has been shown to decrease coronary events in a number of studies. A similar reduction in risk of coronary heart disease death or nonfatal myocardial infarction was observed in the Cholesterol and Recurrent Events (CARE) study [40] and the LIPID study [22-24]. The first occurrence of a nonfatal MI or coronary heart disease death was considered in these analyses [24,41], although other endpoints have been included in other studies [41,42]. In this article, advanced statistical methods were applied to analyze the treatment effect of pravastatin for recurrent myocardial infarctions. The analyses suggest that the treatment effect of pravastatin was significant not only for the first MI event, but also the second MI event during follow-up. Furthermore the magnitude of the treatment effect was similar for first and second events.

There are some limitations in this study. Insufficient statistical power may have limited examination of the importance of the conventional risk factors. The non-significance of the effects of some risk factors for the second MI event could be related to the smaller number of second MI events during follow-up. Greater statistical power can be achieved by expanding the outcome to include other frequently observed endpoints, such as unstable angina or the need for coronary revascularization (percutaneous coronary intervention or coronary artery bypass graft surgery). Another potential limitation is that we did not consider the possibility of informative censoring of a fatal event which would preclude the occurrence of possible future MI events [2,4,39,43-45]. Joint modeling of recurrent events including a possible fatal event is needed in future investigation.

## Conclusions

In conclusion, we found that the application of semi-parametric proportional hazards model to the analysis of recurrent event data is informative and convenient, especially in the estimation of regression coefficients and cumulative risks. The treatment effect of pravastatin was similar on first and subsequent MI events. Important baseline characteristics showed no evidence of different predictive abilities between first and subsequent events.

## Competing interests

The LIPID study was conducted under the auspices of The National Heart Foundation of Australia and funded by Bristol-Myers Squibb. AT, JS, AK, IM and MW were involved in the LIPID study.

## Authors' contributions

JC conducted the data analysis, interpreted the results and led drafting and revision of the manuscript. AF, AK and IM contributed to the planning of statistical analyses and contributed to the writing and revision of the paper. AT, JS, DH and MW were involved in the LIPID study design, follow-up and analysis, and also involved in the conception and design of the present study, interpretation of results and revision and final approval of the manuscript.

## Pre-publication history

The pre-publication history for this paper can be accessed here:

http://www.biomedcentral.com/1471-2288/10/27/prepub
